# Immediate pools of malaria infections at diagnosis combined with targeted deep sequencing accurately quantifies frequency of drug resistance mutations

**DOI:** 10.7717/peerj.11794

**Published:** 2021-11-09

**Authors:** Ozkan Aydemir, Benedicta Mensah, Patrick W. Marsh, Benjamin Abuaku, James Leslie Myers-Hansen, Jeffrey A. Bailey, Anita Ghansah

**Affiliations:** 1 Department of Pathology and Laboratory Medicine, Warren Alpert Medical School, Brown University, Brown University, Providence, RI, United States of America; 2 Department of Parasitology, Noguchi Memorial Institute for Medical Research, University of Ghana, Legon, Accra, Ghana; 3 Department of Epidemiology, Noguchi Memorial Institute for Medical Research, University of Ghana, Legon, Accra, Ghana

**Keywords:** Malaria, *Plasmodium falciparum*, Drug resistance, Molecular inversion probes, Molecular surveillance

## Abstract

Antimalarial resistance surveillance in sub-Saharan Africa is often constrained by logistical and financial challenges limiting its breadth and frequency. At two sites in Ghana, we have piloted a streamlined sample pooling process created immediately by sequential addition of positive malaria cases at the time of diagnostic testing. This streamlined process involving a single tube minimized clinical and laboratory work and provided accurate frequencies of all known drug resistance mutations after high-throughput targeted sequencing using molecular inversion probes. Our study validates this method as a cost-efficient, accurate and highly-scalable approach for drug resistance mutation monitoring that can potentially be applied to other infectious diseases such as tuberculosis.

## Background

The emergence and recent rise of artemisinin resistance in Southeast Asia (Ashley et al., 2014; Cui et al., 2015; Muller, Lu & Von Seidlein, 2019, Tilley et al., 2016) highlights the urgent need for improved surveillance systems, particularly within sub-Saharan Africa, to monitor drug resistance rates and spread. Resistance continues to evolve to other antimalarials including key partner drugs used in artemisinin combination therapies (ACTs) (Djimde et al., 2015). Monitoring the development and spread of resistance is of paramount importance for modeling and optimizing the longevity of current regimens for clinical treatment and public health (Cui et al., 2015; Wesolowski et al., 2018; WHO, 2019). Like other sub-Saharan African countries, a major challenge facing Ghana is how to maximize the quality and extent of surveillance in the context of limited public health resources and a health workforce whose main focus is on providing necessary clinical care and treatment.

While traditional drug treatment efficacy studies are important for the detection of resistance to new antimalarials, once genomic resistance markers have been discovered and validated, molecular surveillance is a viable alternative allowing for broader and more cost-effective monitoring of drug resistance (Apinjoh et al., 2019). Advances in experimental and computational methods of high-throughput sequencing (HTS) have made it both possible and affordable to interrogate numerous genomic loci from thousands of samples (Ghansah et al., 2019). Moreover, the high depth data generated by HTS allows accurate quantification of mixed infections providing detection of single drug resistance mutations at a frequency at or below 1% (Hathaway et al., 2018). Given this highly sensitive and quantitative nature, HTS allows for even more cost-effective sequencing to generate mutation or variant frequencies for the population of interest by sequencing from a pool of collected samples (Cheeseman et al., 2015; Futschik & Schlotterer, 2010; Miller et al., 2017; Ngondi et al., 2017; Taylor et al., 2013; Taylor et al., 2015). Such pooling strategies are particularly efficient for surveillance where the end goal is often to understand the frequency of drug resistance at the regional or clinic level. Sample pooling has allowed for rapid assessments of artemisinin resistance across Africa (Taylor et al., 2015) as well as the spread of new mutations such as the triple mutant phenotype in *pfdhps* underlying sulfadoxine resistance (Ngondi et al., 2017). Although post-collection sample pooling decreases sequencing costs, it still requires extensive time and effort in terms of sample collection in the clinic or field. This is particularly the case of dried blood spots which are notoriously laborious and not easily amenable to automation. Another major issue in malaria-endemic regions in the developing world is that public health efforts are often constrained by limited public health staffing. While often clinical staff are leveraged, this can negatively impact their indispensable role in providing clinical care. Thus, more efficient surveillance methods that can significantly reduce the upfront labor at the collection facility coupled with HTS capacity could lead to greatly expanded surveillance, and provide more temporally and spatially detailed monitoring to inform public health systems.

Here, we report a pilot trial of innovative pooling, immediately at the site of collection, combined with HTS, thereby streamlining the collection and laboratory workflow. From the single tube pools using molecular inversion probes, we generated quantitative targeted HTS for all known drug resistant mutations and screen for novel mutations in important genes such as K13 and show that immediate pools provide accurate results for two sites comparable to the results obtained from individual dried spots, but for a fraction of the effort and cost.

## Methods

### Study sites

Sample collection occurred in two sites in Ghana between June–August 2017: Ewim Polyclinic, Cape-Coast metropolis and Begoro District Hospital in Begoro, Fanteakwa North District. The detailed description of these two study sites is described elsewhere (Mensah et al., 2020). Briefly, Begoro is in the eastern region of Ghana with a forest ecology and high transmission intensity for malaria. Cape coast as its name suggests is located on the coastal belt of Ghana and has low to moderate transmission intensity. Malaria transmission is perennial in both study sites and the two sites are about 245 km apart.

### Study population

The study participants consisted of only symptomatic children aged between six months and fourteen years and tested positive for *P. falciparum* infection by microscopy. Symptomatic children are generally considered to have higher parasitaemia than asymptomatic children.

### Ethics statement

The Institutional Review Board of the Noguchi Memorial Institute for Medical Research (NMIMR) approved this study (#094/16-17). Written informed consent was obtained from adults and from parents or guardians for those under 18 years. In addition, assent was obtained from older children (12–17 years).

### Malaria diagnostics and sampling

For routine malaria testing, patients of all ages suspected of having malaria provided ∼400 µl of finger prick sample for blood smears and for Rapid Diagnostic Tests (RDT) testing. Of this, 50 ul of blood was held in a pipette awaiting test results. Malaria positive samples were ejected into the same 15 mL falcon tube containing 5 mL DNA/RNA Shield (Zymo Research). The growing pool was kept at 4 °C between patients. In addition traditional filter paper dry blood spot DBS (50 µl) was also created per patient and stored desiccated. In all, a pool consisting of an equal volume mixture of 100 sequential *P. falciparum* infections were created by the clinical laboratory technician at each site with minimal extraneous effort. After completion, the pool was transported to Noguchi Memorial Institute for Medical Research (NIMIR) laboratory at 4 °C together with the DBSs and stored at −20 °C.

### DNA extraction and quantification

Genomic DNA was extracted from 200 uL of thoroughly-mixed pooled blood or 3 × 6 mm punches of individual DBSs using the QIAamp kit (QIAGEN^®^) according to the manufacturer’s instructions. The DNA from the pooled blood was eluted  in  200 ul AE buffer, while DNA from filter paper was eluted in 150 uL AE buffer. The quality and quantity of DNA was estimated using the Qubit 2.0 Fluorometer (Thermo Fisher Scientific).

### Control samples

Mixtures of DNA isolated from the laboratory strains 3D7, HB3, 7G8, and DD2 mixed at relative frequencies of 67%, 14%, 13%, and 6%, respectively, were used as positive controls at two concentrations: 29 (low density) and 467 (high density) parasites/ µl, as well as negative controls to test various error rates. These proportions were done to evaluate if our MIP assay and sequencing method can detect all the major and minor alleles present in a pool at different concentrations. Further details on the preparation of the controls and validation process have been described elsewhere (Aydemir et al., 2018).

### Molecular Inversion Probes (MIP) capture, high-throughput sequencing and variant calling

MIPs designed to capture known and candidate drug resistance mutations were used in capture reactions and sequenced on Illumina MiSeq as described (Aydemir et al., 2018). Briefly, each reaction consisting of 5 µl of extracted DNA (1–10 ng) in 10 µl capture reactions containing Phusion DNA polymerase, Ampligase, pooled MIPs, and dNTPs were incubated for 1 h at 60 °C, followed by exonuclease I and II (2 µl) digestion for additional 1 h at 37 °C to destroy template DNA. The entire capture reaction (12 µl) was amplified in a 25 µl PCR reaction with primer combinations to create a uniquely barcoded Illumina sequencing library from each sample. The PCR was performed using a preheated thermocycler with the following steps 98 °C 30 s, 21 cycles (98 °C 10 s, 63 °C 30 s, 68 °C 30 s), 68 °C 2 min, 4 °C hold. Next, all sample barcoded libraries were combined in a single tube (5–10 uL aliquot of each). Remaining adapter primers and dimers were removed and samples were concentrated using SPRI beads and agarose gel purification. The pooled library was sequenced cost-effectively in combination with other libraries with dual indexing using MiSeq Reagent Kit v2 and custom primers to generate 250 base read pairs.

### MIP sequence processing and variant calling

Illumina sequence reads were processed as described elsewhere in detail (Aydemir et al., 2018) with minor changes. Briefly, MIP sequence reads were separated into individual samples based on their Illumina dual barcode. Paired end reads were then stitched together and filtered on expected length (≥30 bp) and on per base quality scores (75% of bases >Q30). Quality filtered stitched sequences were then further separated by MIP target region using the extension and ligation arm sequences to produce a file for each target for each sample. Target sequences were also error corrected based on their unique molecular identifiers (UMIs) by creating a consensus sequence for each specific UMI with multiple read pairs. This UMI redundancy removes a significant proportion of PCR errors that happen in late cycles, polymerase stutter and subsequent sequencing errors (Aydemir et al., 2018). UMI corrected sequences are then further clustered by using the qluster algorithm from SeekDeep to further remove error (Hathaway et al., 2018). Per sample fastq files were created from the UMI corrected, clustered sequences. The variant call set was created by freebayes software (Garrison & Marth, 2012). This variant call set was further filtered to remove low quality sites (QUAL < 1). We set a minimum depth threshold of ≥ 10 UMIs for making a genotype call and a minimum depth threshold of ≥2 UMIs for calling minor variants. All analysis was carried out using MIPTools software (https://github.com/bailey-lab/MIPTools).

### Statistical analysis

All statistical analyses were performed using the statistical software R (version 3.5) (Mensah et al., 2020). The allele fraction was estimated for each sample or pool, and the population frequency of each resistant mutant was estimated as the average allele fraction for that population. For validation of the MIPs, the frequencies of mutations observed were compared with expected frequencies by determining the genotyping rate, accuracy and precision. Frequencies for the known drug resistance mutations within the genes *Pfcrt*, *Pfcytb*, *Pfdhfr*, *Pfdhps*, *Pfmdr1*, *Pfmdr2* and novel variants in *PfK13* of individuals samples and pools were estimated and the Pearson’s correlation coefficient and *p* values estimated. The distribution of mutations was explored by plotting these same mutant combinations for pools and individuals.

## Results

**Figure 1:**
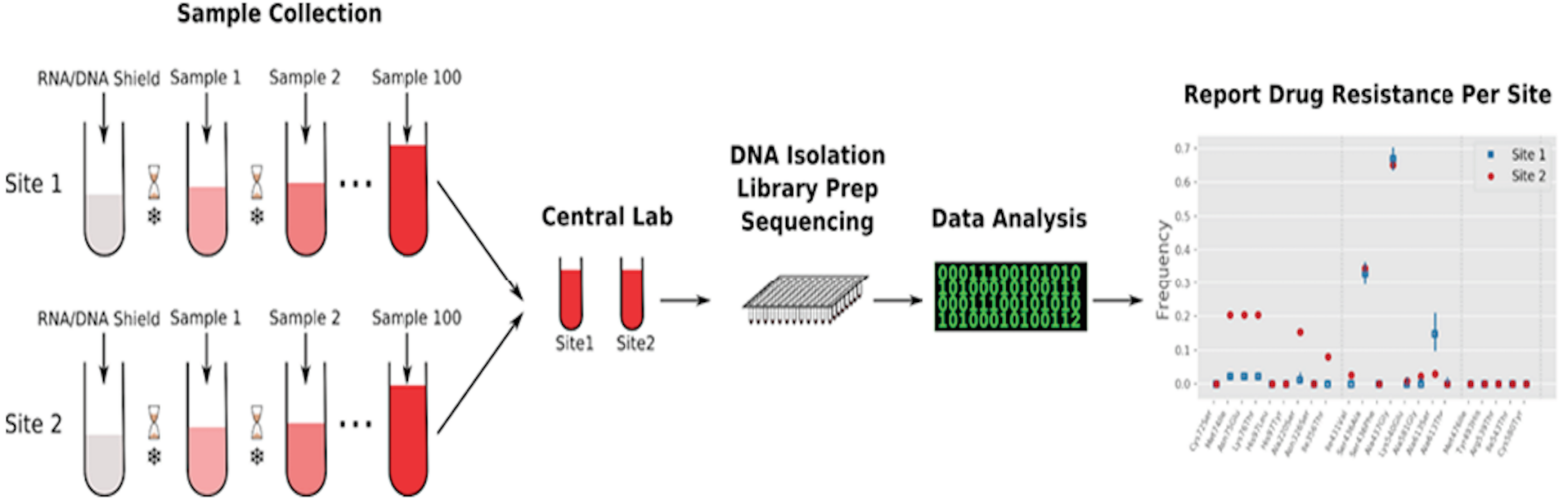
Immediate pooling workflow. From blood held aside during testing, a small amount (50 uL) is ejected into a common tube already containing adequate amount of nucleic acid preservative for the anticipated/planned number of patients. Sequential malaria positive patients are added until a set number of patients and/or set time period is achieved. The final pool is then sent to a central lab for processing to isolate DNA. MIP capture and sequencing is then performed and subsequent bioinformatics analysis yields the frequency of drug resistance mutations for pool.

We designed the immediate pool method to minimize both upfront clinical and downstream laboratory work (Fig. 1, Fig. S1). To test this method, immediate pools of 100 individuals were generated at both Begoro and Cape Coast, each over approximately 3 weeks, by the sequential addition of blood from infected individuals testing positive for malaria. To provide a comparison to individual-level sampling, we also generated separate filter paper dried blood spots (DBSs) from each individual. The work of setting aside a small amount of blood and to be added after testing took minimal effort compared to filter paper processing that required proper drying and storage in desiccant.

After transport to the lab, isolated DNA for pools and individuals were captured using a MIP panel targeting known drug resistance mutations and key genes. In addition, we included 12 positive controls of laboratory strain mixtures and 7 negative controls, to monitor various error rates. Resulting reads from individual samples, site pools (in replicates) and controls were processed and variants called (Table S1).

The combined results of individual captures for each pool generated a total of 20,550 and 108,718 sequences, for Begoro and Cape Coast, respectively. These sequences represented unique capture products based on collapsing any sequences with shared Unique Molecular Identifiers (UMIs). For 82 Begoro and 90 Cape Coast individual samples that yielded MIP sequence, the average was 1,838 and 3,395 (total 150,734 and 305,583), respectively (Table S2). For each of the 28 MIPs in the panel this equated to an average depth per MIP of 2308 UMIs in the pools versus 95 UMIs in the individual samples. High and low density positive controls had on average 9292 and 1235 sequences, respectively. No reads were obtained from negative controls.

Expected within sample allele frequencies in controls closely tracked the observed frequencies (Fig. S2, Table S3). Analysis of 6 high density controls for 38 known drug resistance mutations showed a genotyping rate of 100% (228/228), 99.1% accuracy (226/228), 100% precision (112/112) and 98.2% recall (112/114). 6 low density control samples were genotypable at 87.7% (200/228) with 97% accuracy (194/200), 100% precision (93/93) and 93.9% recall (93/99). No major strain was missed and the majority of missed alleles were at the lowest frequency in the mixture (6%).

**Figure 2:**
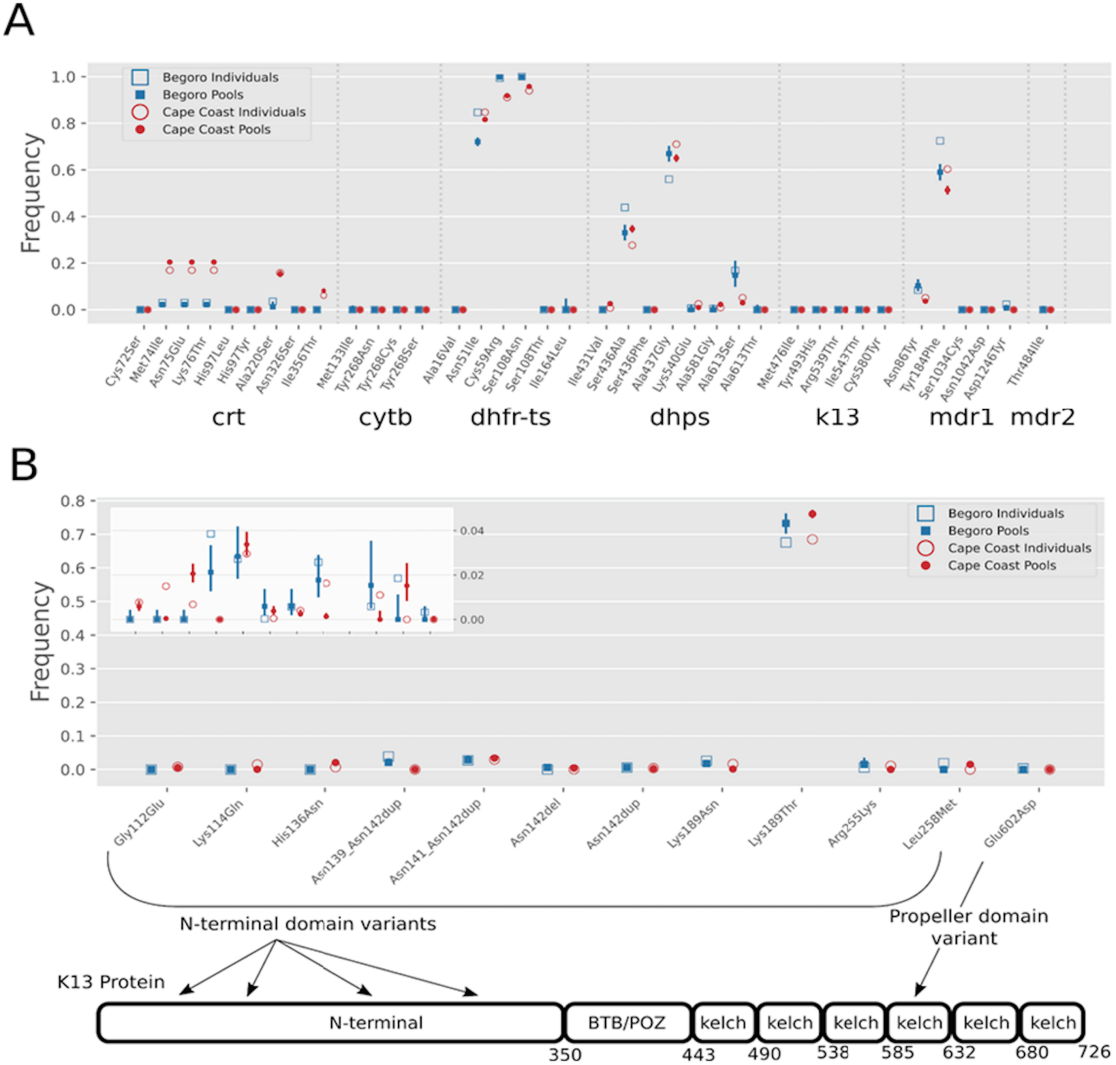
Frequency of drug resistance mutations for Begoro and Cape Coast pools and individuals in key *P. falciparum* genes. (A) Specific known mutations are shown within crt, cytb, dhfr, dhps, k13, mdr1 and mdr2. (B) Overlapping MIPs covering the entire coding sequence of K13 monitoring for new mutations within the gene. All observed variants except Lys189Thr were observed under 4% frequency. Only a single mutation was observed in the propeller domain. The bottom 5% of the plot is shown in the inset to show details of low frequency mutations. Error bars represent 95% CI calculated for pools based on each variants observed count and coverage (UMI number) and beta-binomial distribution..

**Figure 3:**
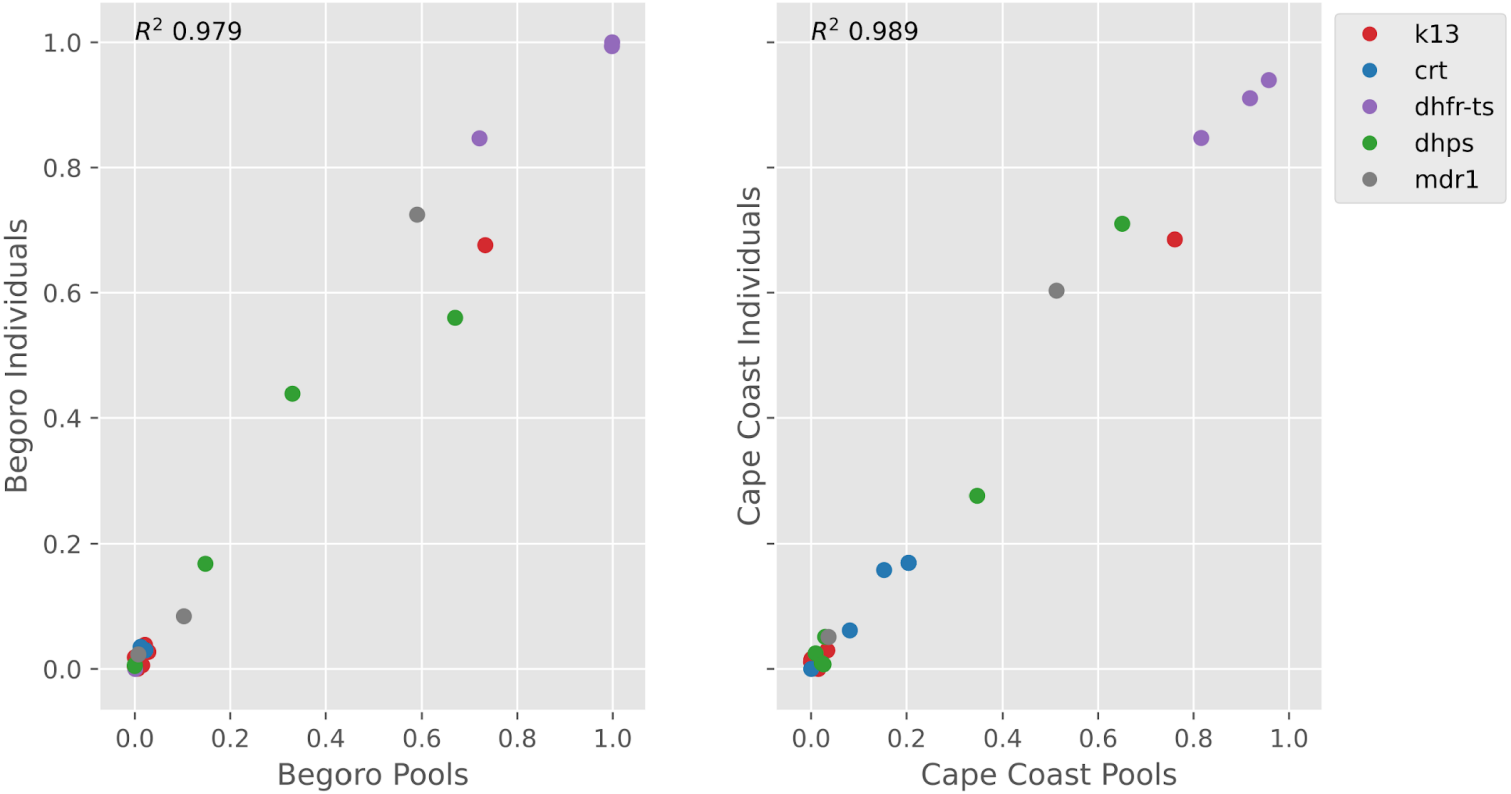
Correlation between population frequency calculated for drug resistance mutations and novel variants in K13 from immediate pools and individual dried blood spots. Mutation frequencies between individuals and pools demonstrate strong correlation *R*^2^ = 0.979 and *R*^2^ = 0.989 respectively for the two sites. Correlation calculation excluded the sites where both individuals and pools had zero frequency.

A central goal of our pilot was to measure how well immediate pools represented frequencies traditionally calculated by combining individual data. Comparing the frequencies of drug resistance mutations and novel variants in K13 within both sites, the pooled and individual sampling correlated extremely well (Fig. 2, Table S4) with Pearson’s R^2^ of 0.979 and 0.989 for Begoro and Cape Coast, respectively (Fig. 3). The two sites showed resistance frequencies consistent with historical surveillance work at these sites including now low levels of chloroquine resistance and absence of actionable K13 mutations (Aydemir et al., 2018). There were 7 mutations which were discordant between the pools and individuals of a site. Five of these were observed in the individuals but missed in the pools and 2 were observed only in the pooled sequences. All discordant mutations had low population allele frequencies; 5 <1% and 2 between 1 and 2%.

## Discussion and Conclusions

The high cost, labor-intense and long turnaround time of current drug resistance monitoring systems in Africa limit resistance monitoring in Ghana and other endemic countries. Here we show that immediate pooling combined with molecular inversion probe sequencing provides effective monitoring generating quantitative results on par with individual level sequencing for significantly less effort and cost minimal loss of sensitivity. We were able to provide accurate population frequency measures of all known drug resistance mutations as well as detection of potential novel variation across the K13 gene.

Our goal was to overcome current limitations of site-based surveillance that most commonly employ DBS, which is labor intensive both for the initial collection given the need to dry and properly desiccate as well as in the laboratory requiring the laborious DNA extraction from filter papers. Here we leverage both pooling as well as newer preservatives to minimize costs and labor. Pooled targeted NGS has emerged as a reasonable alternative for cost reduction and has been applied to quantify resistance alleles (Ngondi et al., 2017; Taylor et al., 2013; Taylor et al., 2015). It also provides a single tube from which to extract and sequence with a cost ∼$125 per pool vs ∼$800 for 100 individual samples.

Thus, immediate pooling coupled with HTS allows for the potential to greatly expand surveillance associated with public health and control efforts. For surveillance work where additional or discarded blood (20–50 uL) can be obtained without written consent, such a collection scheme would require almost no additional effort and standard clinical staff could be employed in larger surveillance schemes with already pooled samples flowing to a centralized lab for sequencing. Thus, we envision that pools could be created at hundreds of facilities multiple times a year or even in continuous fashion. We are expanding the frequency and number of sites in Ghana to provide improved monitoring for the emergence and spread of resistance.

The pooling process is not without its limitations. It by definition does not provide data at an individual level and metrics such as complexity of infection and prevalence, or correlation with clinical data, can not be measured. However, individual-level data could be collected intermittently to generate such metrics along with potential triggers for individual-level investigation at particular sites based on findings in the pool (such as an observation or dramatic shift in mutation frequency). Another limitation is that low frequency variants, occurring in only a few individuals, can be missed within the larger pool, although the number of missing mutations in the pools (5) was not very different from those missing in the individuals (3) in our data set. We think however that this is balanced with the ability to surveil a greater number of sites and a greater number of individuals. In addition, sensitivity could be improved by generating smaller pools (*e.g.*, four 25 individual pools instead of a single 100 individual pool). In scaling surveillance, we were more concerned with specificity and false positives and MIPs afford UMIs that can help clean errors from amplification and sequencing.

Immediate pools may be of more general applicability in infectious disease. Immediate pools may be an excellent way to monitor for other infectious diseases. First, like with *P. falciparum*, it can be used to monitor the population-level frequency of variation of interest such as drug resistance, strain types, or virulence factors in other infections. Second, it could be used to monitor for the occurrence of a particular infection within the pool, *e.g.*, a pool of malaria negative febrile illnesses monitored for viral hemorrhagic diseases. This method may be used for tuberculosis drug resistance monitoring. Also, the pooling method can be very useful for malaria species that cannot be maintained long term in culture like *P. vivax*.

Finally, given it is a pool of human patients human traits of public health importance can be determined for a particular facility, *e.g.*, frequency of sickle cell mutation, or G6PD mutation in areas proposed for primaquine treatment, etc. Overall efficient cost-effective sampling with immediate pooling in health care facilities combined with next generation sequencing may afford and greatly expand surveillance systems and measures in developing areas where information is most needed.

##  Supplemental Information

10.7717/peerj.11794/supp-1 Supplemental Information 1 Read counts for each pool and individual samples

10.7717/peerj.11794/supp-2 Supplemental Information 2 UMI coverage per sample per MIP

10.7717/peerj.11794/supp-3 Supplemental Information 3 Comparison of observed and extected allele frequencies of the controls

10.7717/peerj.11794/supp-4 Supplemental Information 4 Frequencies of mutations in immediate pools and individuals for known mutations and novel variants in K13

10.7717/peerj.11794/supp-5 Supplemental Information 5 Workflow of immediate pooling and sequencing Outlined is the processing occurring with each individual sample to create the immediate pool from confirmed malaria patients as well as downstream DNA isolation, MIP capture and Illumina MiSeq sequencing. Note: only RDT or microscopy positive samples were pooled and a separate pool was created from those who were malaria negative providing additional avenues to pursue in terms of investigating fevers with other origins.

10.7717/peerj.11794/supp-6 Supplemental Information 6 Comparison of observed and expected drug resistance mutation frequency in 12 control mixtures Comparison of observed and expected drug resistance mutation frequency in 12 control mixtures. Mutation frequencies observed in 6 high density and 6 low density control mixtures plotted against the expected frequencies based on mixture components. Horizontal lines marking the expected frequencies

## Additional Information and Declarations

### Data Availability

The following information was supplied regarding data availability: The Plasmodium falciparum raw sequence reads are available at BioProject (SRA-NCBI): PRJNA675948.
