# Supplementary figures and images for: Immediate pools of malaria infections at diagnosis combined with targeted deep sequencing accurately quantifies frequency of drug resistance mutations
Source: PeerJ. 2021 Nov 9;9:e11794. doi: 10.7717/peerj.11794 (PMC8588852; doi:10.7717/peerj.11794)

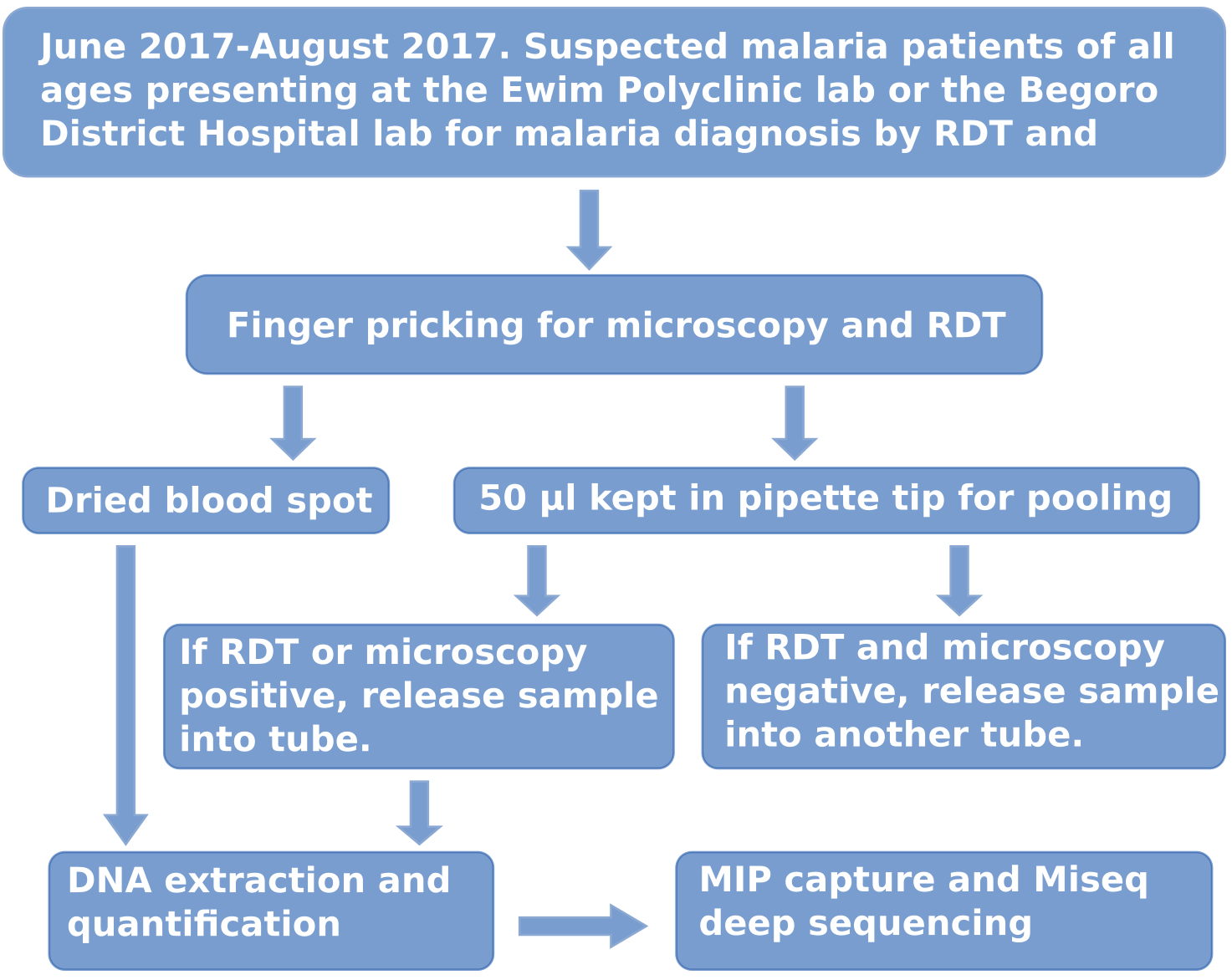

Supplement: Supplemental Information 5 — Outlined is the processing occurring with each individual sample to create the immediate pool from confirmed malaria patients as well as downstream DNA isolation, MIP capture and Illumina MiSeq sequencing. Note: only RDT or microscopy positive samples were pooled and a separate pool was created from those who were malaria negative providing additional avenues to pursue in terms of investigating fevers with other origins. [file peerj-09-11794-s005.png]

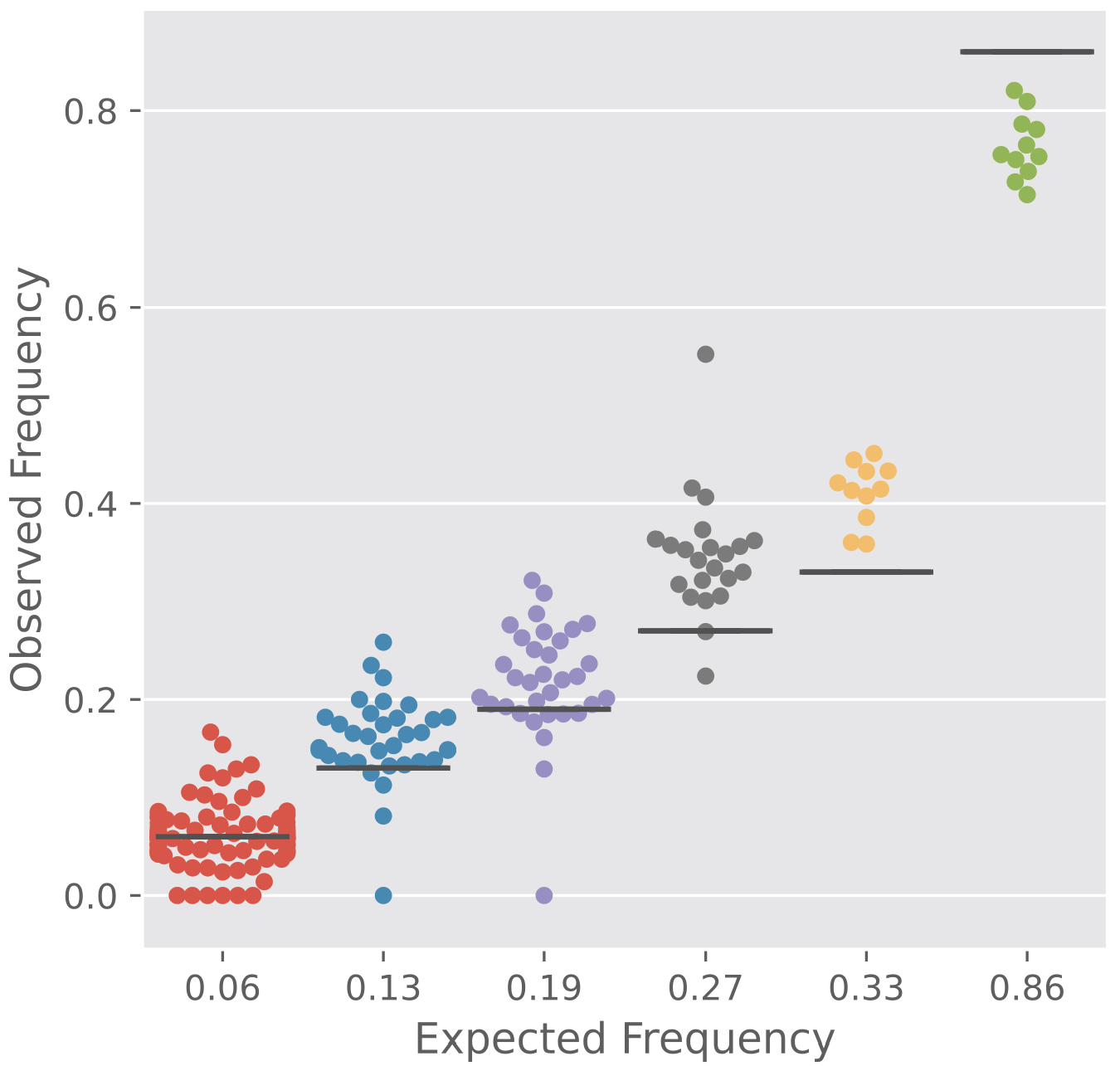

Supplement: Supplemental Information 6 — Comparison of observed and expected drug resistance mutation frequency in 12 control mixtures. Mutation frequencies observed in 6 high density and 6 low density control mixtures plotted against the expected frequencies based on mixture components. Horizontal lines marking the expected frequencies [file peerj-09-11794-s006.png]
